# Effects of Omega‐3 as an adjuvant in the treatment of periodontal disease: A systematic review and meta‐analysis

**DOI:** 10.1002/cre2.736

**Published:** 2023-06-21

**Authors:** Clementine Miroult, Jerome Lasserre, Selena Toma

**Affiliations:** ^1^ Department of Periodontology, Cliniques Universitaires Saint‐Luc Université catholique de Louvain Ottignies‐Louvain‐la‐Neuve Belgium

**Keywords:** clinical attachment loss, docosahexaenoic acid (DHA), eicosapentaenoic acid (EPA), fatty acids, host modulating therapies, Omega‐3, periodontal pocket depth, periodontitis

## Abstract

**Background and Objectives:**

Promoting resolution of inflammation using new classes of lipids mediators has been proposed for the management of inflammatory disease. This systematic review and meta‐analysis aimed to evaluate the benefits of the use of omega‐3 fatty acids as an adjuvant in the nonsurgical treatment of periodontitis.

**Material and Methods:**

The data search was conducted into three main databases: PubMed, Embase, and Cochrane. The search equation was built around the PICO framework in which the population was constituted by human adults suffering from chronic periodontitis that had to be treated with conventional SRP with the adjunction of omega‐3 fatty acids (I) or without the adjunction of omega‐3 fatty acids (C), with, as a first outcome the probing pocket depth reduction (PPD) and as a second outcome the clinical attachment loss reduction (CAL). Risk of bias within studies was evaluated for each included study using the Cochrane collaboration tool for randomized studies (RoB Tool). A meta‐analysis was performed using REVMAN 5.3.

**Results:**

After a global search, 117 studies were selected but only seven of them were eligible for the systematic review and meta‐analysis. Six out of seven studies showed a significantly better PPD reduction in the omega‐3 fatty acids group compared to the control group and five out of seven studies showed a significantly better CAL reduction in the omega‐3 fatty acids group compared to the control group. The meta‐analysis showed a statistically significant difference for PPD reduction (SMD: −0.78 [95% CI: −1.02, −0.54, *p* < .0001]) and CAL reduction (SMD: −0.80 [95% CI: −1.04, −0.56, *p* < .0001]) in favor of the test group.

**Conclusion:**

After scaling and root planning, PPD reduction and CAL reduction were observed in both control and test groups, but with statistically significant better values for the omega‐3 fatty acids group. Patients suffering from periodontitis could benefit from the use of omega‐3 fatty acids to increase the effectiveness of a nonsurgical treatment.

## 1 INTRODUCTION

Periodontal inflammation is initiated by the presence and accumulation of bacterial pathogens, but the progression of the disease is mainly due to a dysbiosis into the microbiome caused by the inflammatory response in susceptible individuals (Van Dyke & Serhan, 2003; Michalowicz et al., 2000). While the etiology of the disease is caused by biofilm, most tissue destruction is caused by the host inflammatory response. When an inflammatory event is terminated, the complete clearance of invading leukocytes from the inflammation site is required to allow homeostasis. Those mechanisms have created some interest in therapies aiming at the host response cascade (Van Dyke & Serhan, 2003). Those new therapies are known as host modulating therapies (HMT's), they will impact the host response by lowering the excess amount of arachidonic acids (AA) metabolites and MMPs produced through the inflammatory response.

Regarding this perspective, interest has raised concerning Omega‐3 fatty acids. Omega‐3 fatty acids are long‐chain essential polyunsaturated fatty acids or PUFA's. They can be found in various foods such as fish oil, linseed, and walnut oil (Tur, Bibiloni, et al., 2012). Omega‐3 PUFA's can be metabolized into eicosapentaenoic acid (EPA) and docosahexaenoic acid (DHA). These families of anti‐inflammatory molecules are not immunosuppressive, instead they will work by activating proresolving mechanisms to achieve homeostasis. Moreover, EPA and DHA can also synthesize specialized proresolving mediators (SPM). EPA will produces resolvins (RVE_1_, RVE_2_) and DHA will produce protectins (PD_1_) and maresins (MaR) (Serhan et al., 2002). These SPM's will limit neutrophil (PMN) recruitment at the inflammatory site but also stimulate clearance of apoptotic macrophages and bacterial killing. Resolvins (RvD_s_) can also be aspirin‐triggered, leading to an epimeric form with an increased half‐life (Dalli et al., 2013). In vivo, resolvin E_1_ demonstrates a protective effect against inflammation in both gingival tissue and bone in animal models (Hasturk et al., 2006). Resolvins and protectins also have antioxidative properties: a study conducted on rats showed an upregulation of enzymes catalase and superoxide‐dysmutase when those rats were fed with omega‐3 fatty acids (Kesavalu et al., 2007). SPM's will activate the proresolving circuit and will promote periodontal healing and even periodontal regeneration (Van Dyke, 2017). Besides periodontal disease, anti‐inflammatory and antioxidative properties exhibited by SPM's (Campan et al., 1997; Eberhard et al., 2002) could also be protective in various diseases including rheumatoid arthritis, cystic fibrosis, ulcerative colitis, asthma, atherosclerosis, cancer, cardiovascular disease but also in periodontitis (Serhan et al., 2008). There are no actual recommendations about the adjunctive use of omega‐3 in periodontal health. When treating patients with risk factors such as diabetes or smoking habits, periodontal healing could be more difficult to achieve, especially in deep pockets and on multirooted teeth (Tomasi et al., 2007). In those cases, the additional use of omega‐3 could be even more favorable. The fact that PUFA's are relatively easy to find in diet, with no adverse effects on systemic health shows that it seems primordial to assess their effectiveness in periodontal therapy. It this study, it is hypothesized that omega‐3 could be beneficial in the treatment of periodontitis. To test this hypothesis, a systematic review of the actual literature and a meta‐analysis will be conducted.

## 2 MATERIAL AND METHODS

### 2.1 Search strategy

This review was conducted in accordance with the Cochrane Handbook for Systematic Reviews of Interventions (Higgins JPT 2019) and reported according to the PRISMA guidelines (Liberati et al., 2009). In April 2020, relevant studies were identified through electronic search in three main databases from the earliest records until January 2021. The following databases were searched: PubMed, Embase, and the Cochrane Library. The focus question was “Could Omega 3 intake improve nonsurgical treatment outcome in patients with chronic periodontitis?”. The search terms included “periodontitis,” “periodontal disease,” and “therapy,” “treatment” as key words for the disease and the treatment and “omega 3,” “fatty acids,” “fish oil,” “polyinsaturated fatty acids” for the intervention. The full search strategy is available in Supporting Information: Appendix S1.

### 2.2 Eligibility criteria

Studies were assessed for eligibility based on the Pico criterias.

#### 2.2.1 Participants

Studies including adults (>18 years old) patients suffering from chronic periodontitis. Patients suffering from systemic diseases that could eventually alter periodontal status and healing were also excluded.

#### 2.2.2 Intervention

The treatment must involve the initial treatment, scaling, and root planning (SRP) with the adjunction of omega‐3 afterwards with an available dosage. Studies involving surgical treatment of periodontitis were excluded. Also, studies administrating antibiotics in adjunction to omega‐3 were excluded.

#### 2.2.3 Comparison

SRP alone or with placebo.

#### 2.2.4 Outcome

Studies' outcomes variables must be available in both groups in order to be eligible. The first outcomes evaluated was the “Probing Pocket Depth” or PPD. The second outcome was the “Clinical Attachment Loss” or CAL.

#### 2.2.5 Study type

Randomized controlled trials (RCT) were eligible for inclusion. Noninterventional studies were excluded. A minimum follow‐up of 1 month was required to assess the main clinical parameters of periodontal healing

### 2.3 Study selection

Databases research was conducted by one reviewer, then all citations and abstract results were exported into a reference management software (EndNote X9^©^). Reviewers (Selena Toma and Clémentine Miroult) screened titles and abstracts for eligibility. Screened abstracts that were eligible for inclusion had their full text assessed by the two reviewers. Reasons for exclusion were also reported. When full text articles were not available online, authors were contacted by e‐mail. Cohen's *κ* score for interrater reliability was also calculated. Finally, when a discordance occurred, a third reviewer was available to find a consensus.

### 2.4 Data extraction process

The extracted data included: the first author's name, country of origin, the year of publication, the diagnostic criteria for periodontitis, the number of control and test patients per study arm, type of omega 3, dosage of the omega 3 and duration of omega 3 intake. The continuous variables PPD and CAL of the included studies were analyzed as individual values at the end of each study. Data were analyzed using Review Manager software (version 5.3).

### 2.5 Risk of bias assessment in individual studies

Quality assessment at a study level was performed by each reviewer, independently, by using the online RoB 2.0 tool (Risk of Bias assessment for Randomized trials) (Sterne et al., 2019). Studies were assessed on six criteria that could lead to potential sources of bias: sequence generation, allocation concealment, blinding of participants and personnel, blinding of outcome accessors, incomplete outcome data, selective outcome reporting, and other bias. Each of the criteria was judged at low risk, high risk, or unclear risk. If there was a disagreement it would be solved after discussion between the two reviewers. A second risk of bias assessment was performed on an outcome level, evaluating potential sources of bias arising from differences in outcome measures between studies.

### 2.6 Risk of bias assessment across studies

Risk of bias across studies that may affect the cumulative evidence of the review were assessed by both reviewers by using the online GRADE tool (Kruse et al., 2020). According to GRADE terminology, the quality of the overall body of evidence for each outcome was: “high certainty” for high‐quality evidence; we are very confident that the true effect lies close to that of the estimate of the effect. “Moderate certainty” for moderate‐quality evidence; we are moderately confident in the effect estimate: the true effect is likely to be close to the estimate of the effect, but there is a possibility that it is substantially different. “May/may not” for low‐quality evidence; our confidence in the effect estimate is limited; the true effect may be substantially different from the estimate of the effect and, “large uncertainty” for very low‐quality evidence; we have very little confidence in the effect estimate: the true effect is likely to be substantially different from the estimate of the effect. RCT's studies are usually classified as high quality but this can be reduced for some reasons as the methodological design, the study quality, consistency, and directness.

### 2.7 Statistical analysis and data synthesis

A first analysis including all studies in a single pool was performed. The estimates of the intervention effects (SMD, standard mean difference) were expressed in millimeters with a classic 95% confidence intervals (CI's). The inverse variance method was used for the fixed‐effect models. To evaluate heterogeneity *χ* ^2^ tests were performed. Values ranging under 25% were considered to be low heterogeneity, values between 25% and 50% were considered moderate, and values higher than 50% were considered as high heterogeneity (*Higgins JPT 2019*). When heterogeneity was low or medium, the random effect model evaluated the variance components in the presence of heterogeneity (*p* < .10) rather than the fixed‐effect model. Finally, a funnel plot comprising SMD and standard error (SE) was drawn for each outcome variable to assess a possible publication bias across selected studies. Studies outside the confidence interval area may indicate possible publication bias. The effect was considered statistically significant for *p* ≤ .05.

### 2.8 Additional analysis

After the first analysis including all studies in a single pool, a subgroup analysis only including omega‐3 + aspirin was also conducted for each outcome. This subgroup analysis was conducted to emphasize the potential effect of aspirin in conjunction with omega‐3 intake.

## 3 RESULTS

### 3.1 Study selection and description

A total of 117 studies were identified through online searches and 43 duplicates had to be removed.

**Figure 1:**
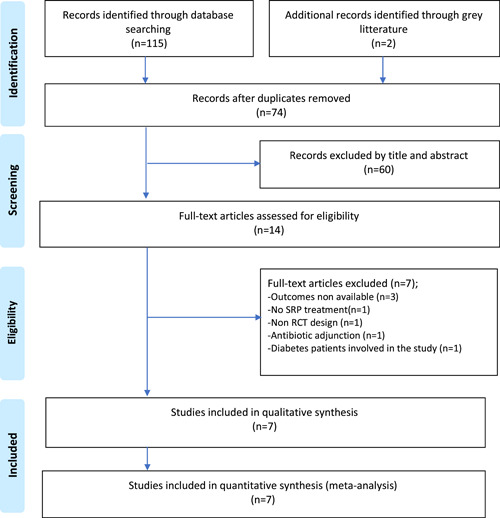
Flowchart demonstrating the study selection process.

Afterwards, 74 records were screened through their title and abstract and 60 of them were excluded because they did not respond to the inclusion criteria. Therefore, 14 studies were eligible for full text reading. After applying eligibility criteria, six studies had to be excluded. Finally, seven studies were included in the qualitative and quantitative analysis as represented in Figure 1. Cohen's *κ* scores were calculated, and the results were >80%, demonstrating a good interexaminer reliability.

### 3.2 Studies characteristics

Figure 2 lists in detail the characteristics of each included study. The studies were all written in English and conducted between 2010 and 2020. All of them were conducted for comparison between a test and a control group at a single center. The follow‐up period ranged from 6 weeks to 6 months. This review included 299 participants which included 149 controls and 150 test patients. None of the studies included patients suffering from systemic diseases. One of the studies only included postmenopausal women (Elgendy & Kazem, 2018) and one study had one smoker in each *group* (Martinez et al., 2014). Scaling and root planning were done traditionally in each study including the use of manual curettes and ultra‐sonic devices. All the test patients were given omega‐3 in capsules but with different EPA/DHA dosages. Two studies also included the ingestion of aspirin, 80 mg once a day, while taking the omega‐3 capsule (El‐Sharkawy et al., 2010; Farhad et al., 2014).

Figure 2Studies characteristics.

**Figure:**
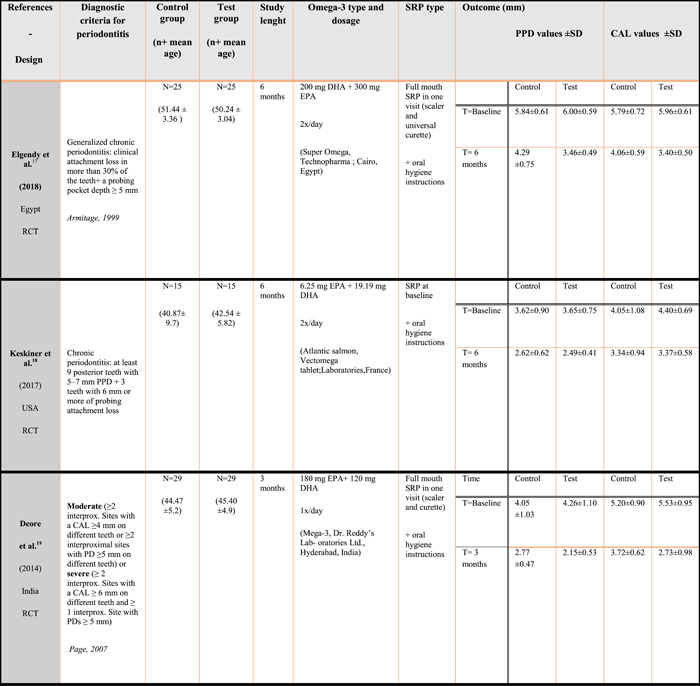

**Figure:**
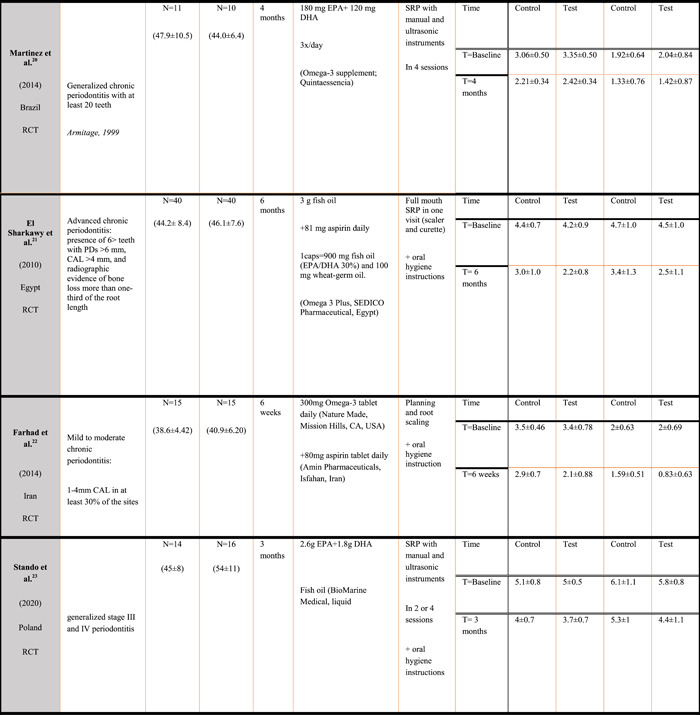

Elgendy and Kazem (2018): In this RCT study, the goal was to investigate the changes in periodontal parameters and superoxide dismutase after complete SRP with or without the adjunction of omega‐3. This study included 50 postmenopausal women suffering from chronic periodontitis. The conclusion was that the test group achieved a better mean PPD reduction and a better mean CAL gain compared to the control group, as well as a greater increase in superoxide dismutase (SOD) activity (*p* < .01) compared to SRP alone.

Keskiner et al. (2017): This RCT's aim was to test the effect of omega‐3 on chronic inflammatory diseases including periodontitis. They tested 15 healthy subjects with low doses of PUFA's and 15 subjects with placebo. They also took saliva samples and analyzed them for tumor necrosis factor‐α (TNF‐α) and SOD levels. The results showed some significant changes in clinical parameters in both groups but there was no significant difference between the test and control group at the end. The conclusion showed that low‐dose omega‐3 supplementation did not have any impact on clinical parameters in patients suffering from chronic periodontitis.

Deore et al. (2014): This RCT examined if omega‐3 could reduce clinical inflammation parameters. Two groups, including 30 patients each, were treated traditionally with SRP with omega 3 or with a placebo. They evaluated the plaque index, the gingival index, the oral hygiene index, the bleeding on probing, the probing pocket depth, the clinical attachment level, and the serum CRP levels. When comparing the test group to the control group, a significantly greater PPD, CAL, GI, and BOP was noticed for the test group. There were no changes in the PI and serum CRP levels.

Martinez et al. (2014): This RCT included 11 control and 10 test patients that all suffered from chronic periodontitis. Test patients received omega‐3 capsules three times a day for 4 months (900 mg/day). Periodontal parameters such as PPD, PI, CAL, and BOP were measured at baseline and 4 months. Also, DHA and EPA serum levels were also evaluated at the same time. The conclusion was that the adjunction of omega‐3 with SRP does not offer a significant difference when compared to treatment without omega‐3.

El‐Sharkawy et al. (2010): In this study, they chose to evaluate the combination of omega‐3 with low‐dose aspirin when treating chronic periodontitis. The study included 80 patients, the test group received omega 3 and aspirin (81 mg/day), control group only received a placebo. An evaluation of the clinical parameters (PPD, CAL, BOP, PI, GI) was carried out at baseline, 3 months and 6 months. Nuclear factor‐κB ligand (RANKL) and matrix metallproteinase‐8 (MMP‐8) values were also part of the study through saliva analyses in each group. At the end, they concluded that the group that received omega‐3 showed a significant reduction of PD and CAL when compared to the control group. Also, RANKL and MMP‐8 were also significantly lower in the test group.

Farhad et al. (2014): This RCT study compares the efficacy of omega‐3 and low‐dose aspirin to doxycycline and placebo in the treatment of periodontitis. The study included 45 patients that were randomly assigned to one of the three groups after receiving SRP treatment. Clinical parameters were evaluated at baseline, then at 6 weeks. The conclusion was that there was a significant decrease of the main clinical parameters (PPD, CAL, BOP) in the test groups compared to the placebo and that this reduction was significantly greater for the omega‐3 group.

Stando et al. 2020 (Higgins et al., 2019): In this RCT they aimed to evaluate the effect of omega‐3 supplementation in patients with stage III and IV periodontitis. This study included 30 patients, treated with scaling and root planning. Clinical parameters such as PPD, CAL, and BOP were evaluated at baseline and at 3 months after initial therapy. Salivary samples were also taken to evaluate interleukin 8 (IL‐8), IL‐10, and IL‐17 levels. At the end of the study, a statistically significant reduction in PPD, CAL, and BOP were noted in the test group. Moreover, the levels of proinflammatory cytokines/chemokines IL‐8 and IL‐17 were markedly lower in the omega‐3 group.

### 3.3 Risk of bias within studies

#### 3.3.1 On a study level

**Figure 3:**
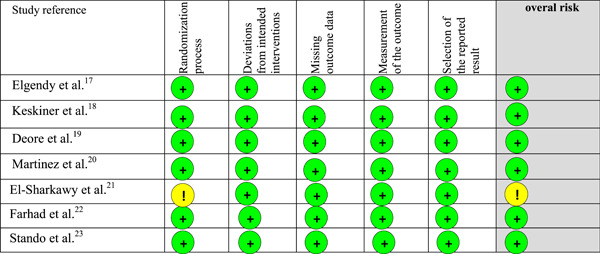
Risk of bias within studies on a study level (Rob‐Tool).

Six of seven studies were considered low risk of bias, and therefore of high quality. One study (Heitz‐Mayfield & Lang, 2013) showed some concern in the overall risk of bias because the first patient enrolled was assessed to a group by toin‐coss, followed by the second patient that was immediately put in the opposite group. If the blinding had to be removed for a reason, it could have led to the loss of double blindness. Figure 3 illustrates the risk of bias for each study.

#### 3.3.2 On an outcome level

##### Bias related to disease severity

All the participants suffered from chronic periodontitis with different degrees of severity. In one study (Heo et al., 2022) only patients with mild to moderate periodontitis were included. Other studies (Hasturk et al., 2006) included patients regardless of their disease severity, and one study (Harris, 2007) included patients with periodontitis ranging from moderate to severe. Finally, three studies (Elwakeel & Hazaa, 2015; Farhad et al., 2014; Higgins et al., 2019) did include patients with PPD values above >5 mm. Literature shows that initially deeper pockets (>6 mm) tends to show more PPD reduction and more attachment gain after SRP when compared to shallow pockets (<6 mm) (Kesavalu et al., 2007). The different disease states of each included study marked by a difference in initial probing pocket depth could possibly lead to a bias in terms of healing potential between studies.

##### Bias related to the omega‐3 ingestion

All the included studies had a clear and detailed indication of their respective omega‐3 dosage. Due to the different dosages in each study, ranging from 50 mg/day (Farhad et al., 2014) to 4400 mg/day (Higgins et al., 2019) there is a possible dose‐related bias for the measured outcomes.

##### Bias related to periodontal therapy

Included studies did use different protocols for scaling and root planning. Full mouth disinfection in one session was performed in three studies (Elwakeel & Hazaa, 2015; Harris, 2007; Heitz‐Mayfield & Lang, 2013) Two other studies had a quadrant‐wise approach (Hasturk et al., 2006; Higgins et al., 2019) and two studies did not mention their exact SRP protocol (Farhad et al., 2014; Heo et al., 2022).

##### Bias related to the follow‐up period

In this review, only three studies had a follow‐up period up to 6 months (Elwakeel & Hazaa, 2015; Farhad et al., 2014; Heitz‐Mayfield & Lang, 2013). Three studies included measures up to 3 months (Harris, 2007; Hasturk et al., 2006; Higgins et al., 2019). After nonsurgical therapy, periodontal healing shows major changes in PPD and CAL reduction between 1 and 3 months (Kesavalu et al., 2007; Kruse et al., 2020). One study had only a 6‐week follow‐up (Heo et al., 2022). A too early re‐evaluation of these clinical parameters could be prejudicial for the healing of the gingiva and could also lead to misinterpreted values.

### 3.4 Statistical analysis and synthesis of results

Seven studies were included in the meta‐analysis (Elwakeel & Hazaa, 2015; Farhad et al., 2014; Harris, 2007; Hasturk et al., 2006; Heitz‐Mayfield & Lang, 2013; Heo et al., 2022; Higgins et al., 2019). The meta‐analysis included the mean values for the final mean of each outcome (PPD and CAL). Different time points ranging from 1 to 6 months were measured in the selected studies. To compare those studies equitably the meta‐analysis included the last measures taken from the last visit at the end of each study. Also, subgroup analyses including studies using aspirin in adjunction with the Omega‐3 was also calculated (Heitz‐Mayfield & Lang, 2013; Heo et al., 2022)

#### 3.4.1 Meta‐analysis for probing pocket depth

##### Pooled results

Seven clinical trials evaluating the influence of periodontal therapy plus omega‐3 in the mean of PPD, in millimeters per tooth, were included. Overall, the pooled results for effect for the omega‐3 group (*n* = 150) was statistically superior to the results for the control group (*n* = 149). The SMD value was −0.78 (95% CI: −1.02, −0.54, *p* < .0001). The estimate was associated with a considerable level of heterogeneity (*I* ^2^ = 67%) and the test for heterogeneity was statistically significant (*p* = .006). This high heterogeneity was explored, and one study (Hasturk et al., 2006) did not fit into the funnel plot. This study had the smallest pool of patients and this could explain a publication bias. When conducting a second analysis ignoring this study, heterogeneity values dropped to *I* ^2^ = 36%, *p* = .17 with SMD values of −0.89 (95% CI: −1.14, −0.64, *p* < .0001). To not overestimate the effect of the intervention the study from Martinez et al., was included as well.

##### Subgroups analysis

**Figure 4:**
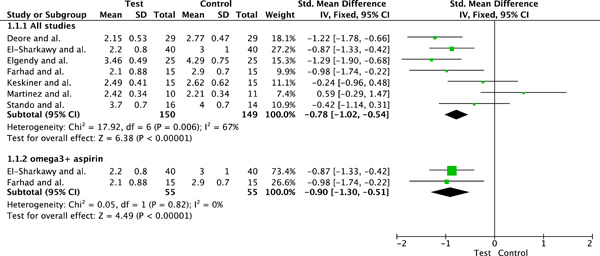
Meta‐analysis for the outcome “probing pocket depth” at end point and subgroup analysis.

Subgroup analyses including only the two studies (Heitz‐Mayfield & Lang, 2013; Heo et al., 2022) using aspirin in conjunction with the omega‐3 were also conducted. Studies using aspirin in addition to omega‐3 (*n* = 55) presented greater PPD reduction values when subgroup analyses were conducted: SMD: −0.90 (95% CI: −1.30, −0.5, *p* < .0001), *I* ^2^ = 0%, *I* ^2^ *p* = .82. Figure 4 illustrates the meta‐analysis results.

#### 3.4.2 Meta‐analysis for clinical attachment loss

##### Pooled results

Seven clinical trials evaluating the influence of periodontal therapy plus omega‐3 in the mean of CAL, in millimeters per tooth, were included. The pooled heterogeneity was considerable (*I* ^2^ = 59%, *p* = .02). This high heterogeneity was explored, and two studies (Farhad et al., 2014; Hasturk et al., 2006) did not fit into the funnel plot. One of the studies has the smallest pool of patients and this could explain a publication bias. The other study had very low quantities of omega‐3 ingested compared to other studies. When conducting a second analysis ignoring those two studies, heterogeneity values dropped to *I* ^2^ = 0%, *p* = .60. To not overestimate the effect of the intervention those studies were finally included in the meta‐analysis as well. Patients treated with SRP plus omega‐3 (*n* = 150) presented a final mean of CAL smaller than patients treated with SRP only (*n* = 149), SMD: −0.80 (95% CI: −1.04, −0.56, *p* < .00001), (*I* ^2^ = 59%, *I* ^2^ *p* = .001).

##### Subgroup analysis

**Figure 5:**
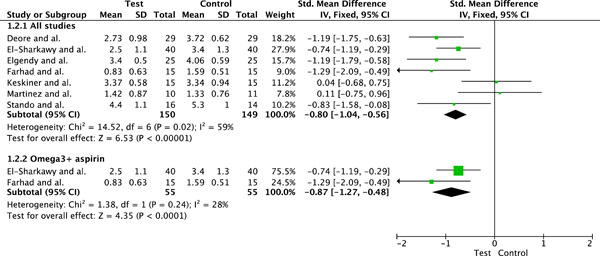
Meta‐analysis for the outcome “clinical attachment loss” at end point and subgroup analysis. CI, confidence interval.

Subgroup analyses including only the two studies (Heitz‐Mayfield & Lang, 2013; Heo et al., 2022) using aspirin in conjunction with the omega‐3 were also conducted. Studies using aspirin in addition to omega‐3 (*n* = 55) presented greater CAL reduction values when subgroup analyses were conducted SMD: −0.87 (95% CI: −1.27, −0.48, *p* < .0001), (*I* ^2^ = 28%, *I* ^2^ *p* = .24). Figure 5 illustrates the meta‐analysis for the clinical attachment loss outcome.

### 3.5 Level of evidence: GRADE tool

**Figure 6:**
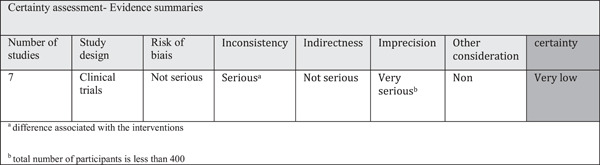
Level of evidence (GRADE tool).

Meta‐analysis including randomized clinical trials do have the best initial quality of a body of evidence (Liberati et al., 2009). First, the inconsistency across studies was considered as serious because of the differences associated with the intervention; the global heterogeneity for each outcome was considered as substantial (*I* ^2^ > 60%). That lack of homogeneity can be explained by the use of different dosage of omega‐3 among studies, with a larger effect size with higher dosages but also by the different durations of follow‐up. There was no indirectness issue, this item was considered as not serious. The imprecision was considered as very serious because of the small number of participants. When a study includes less than 400 subjects, review authors and guideline developers should consider rating down for imprecision (El‐Sharkawy et al., 2010). The overall certainty for this study was considered very low. Figure 6 illustrates the evidence summary for certainty assessment.

## 4 DISCUSSION

### 4.1 Summary of key findings

This systematic review evaluated the hypothesis that omega‐3 fatty acids could be used as an effective adjunct to the nonsurgical periodontal therapy. Seven clinical trials were included in this systematic review and meta‐analysis. Six of them were in favor of the use of Omega‐3 as an adjuvant to the nonsurgical treatment of chronic periodontitis when evaluating PPD. Regarding CAL, only five of them showed better values in the experimental group. The meta‐analysis showed an overall improvement in both PPD and CAL values with an effect measure of −0.78 mm for the PPD outcome and −0.80 mm for the CAL outcome. The included studies that did not show positive results had some limitations: the study from Martinez et al. could have been biased by its small sample of patients (*n* = 10/11). It was also the only study that did not mention instructions for oral hygiene as part of the initial treatment. A second study (Farhad et al., 2014) that did not show an improvement in both parameters, this study was based on a low‐dose approach for the omega‐3 dosage. That dose effect could possibly explain the lack of results in this study. However, dose‐effect‐related results are also a proof of directiveness when evaluating the efficacy of a drug in studies which is beneficial for the overall quality of evidence of the review. Finally, a subgroup analyses for two studies (Heitz‐Mayfield & Lang, 2013; Heo et al., 2022) including aspirin in combination with omega‐3 was conducted. When comparing the results of this subgroup analysis, the estimation for the effect value was greater for both outcomes compared to the pooled results. Aspirin triggered SPM's are proved to be much potent than their native form, they also seem to have an increased half‐life which corroborate with the good results obtained for those subgroup analyses (Campan et al., 1997; Martinez et al., 2014; Michalowicz et al., 2000). However, due to the small number of studies in those subgroup analyses those conclusions must be taken with caution.

### 4.2 Limitations and implications in the context of evidence

The limitation of this systematic review itself was characterized by the lack of homogeneity among the included studies. This lack of homogeneity could be explained by different issues. First, different baseline severities of periodontitis were compared, with different definitions for periodontitis. The included studies were conducted on different time periods, from 2010 to 2020, during those early years a clear consensus for the definition of periodontitis was not established yet. The new classification for periodontal diseases (Morrison et al., 1980) could possibly decrease this bias.

A previous systematic review and meta‐analysis had the same aim and protocol than ours (Kruse et al., 2020). It included six studies with three of them including patients with diabetes but also studies involving surgical treatment of periodontitis (Serhan et al., 2008, 2002). Unfortunately, it seems that they could not obtain outcome measures from one study (Keskiner et al.,^2017^). Finally, they could not include two studies that were not published yet (Higgins et al., 2019). Even though their selection criteria were not the same than those selected for this review, their final conclusions are in accordance with those obtained in this study. Another meta‐analysis (Stańdo et al., 2020) was also published recently, it included 13 studies and evaluated the PPD reduction, CAL gain, and the BOP reduction with the same criteria than our study, except that it included gingivitis patients. After a 6 months follow up the PPD reduction was −0.81 mm and the CAL gain was −0.77 mm, these results are similar to ours. Moreover, a narrative review (Stark et al., 2016) did also bring positive results when assessing the use of omega‐3 in controlling inflammatory processes in human and animals studies. This review also concluded that the adjunction of aspirin with omega‐3 was of great benefits. In contrast, another recent review (Sterne et al., 2019) that also evaluated omega‐3 adjunction in periodontal disease could not show any positive effects on clinical parameters. This review had several limitations because it only included three studies, with only one involving SRP treatment. Scaling and root planning are considered as the gold standard for the treatment of periodontitis (Sun et al., 2007). Nevertheless, it has its own limitations inherent to the pathology itself. As for other inflammatory diseases, omega‐3 intake could provide a public health measure to prevent inflammatory process in population at risk for those diseases. The omega‐3 intake in most population is insufficient to provide the minimal EPA/DHA levels recommended by the World Health Organization (250 mg EPA and DHA/day). In the Belgian population, DHA and EPA intake are considered to be low (<200 mg/day) (Tomasi et al., 2007) this emphasize even more the relevance of this study. Finally, the eventual risk of long‐term omega‐3 intake is considered low. A cardiologist expert opinion did not outweigh any theoretical risks for increased bleeding when taking omega‐3 supplementation and concluded that it was risk‐free, even in patients taking antiplatelet and anticoagulant medication (Tur et al., 2012). Additionally, due to the various antibiotics side effects but also their increasing bacterial resistances, alternatives therapies as HMT's are crucially needed. Regarding this study's positive results on clinical periodontal parameters and the safety and inexpensiveness of omega‐3 intake, it should be encouraged to integrate them into the initial treatment of periodontitis.

## 5 CONCLUSION

This systematic review and meta‐analysis suggested that the adjunction of omega‐3 PUFA's in the initial periodontal therapy could lead to a statistically significant improvement of the PPD and CAL outcomes in patients with chronic periodontitis compared to initial periodontal therapy without omega‐3 PUFA's adjunction.

## AUTHOR CONTRIBUTIONS

*Conceived and designed the analysis, collected the data, contributed data or analysis tools, performed the analysis, wrote the paper*: Clementine Miroult. *Conceived and designed the analysis, contributed data or analysis tools, performed the analysis*: Jerome Lasserre. *Conceived and designed the analysis, contributed data or analysis tools, performed the analysis*: Selena Toma.

## CONFLICT OF INTERESTS STATEMENT

The authors declare no conflict of interests.

6

## Supporting information

Supporting information.

## Data Availability

Data sharing is not applicable to this article as no new data were created or analyzed in this study.

## References

[cre2736-bib-0005] Campan, P. , Planchand, P. O. , & Duran, D. (1997). Pilot study on n‐3 polyunsaturated fatty acids in the treatment of human experimental gingivitis. Journal of Clinical Periodontology, 24(12), 907–913. 9442428 10.1111/j.1600-051x.1997.tb01210.x

[cre2736-bib-0008] Dalli, J. , Winkler, J. W. , Colas, R. A. , Arnardottir, H. , Cheng, C. Y. C. , Chiang, N. , Petasis, N. A. , & Serhan, C. N. (2013). Resolvin D3 and aspirin‐triggered resolvin D3 are potent immunoresolvents. Chemistry & Biology, 20(2), 188–201. 23438748 10.1016/j.chembiol.2012.11.010 PMC3583372

[cre2736-bib-0009] Deore, G. D. , Gurav, A. N. , Patil, R. , Shete, A. R. , NaikTari, R. S. , & Inamdar, S. P. (2014). Omega 3 fatty acids as a host modulator in chronic periodontitis patients: A randomised, double‐blind, palcebo‐controlled, clinical trial. Journal of Periodontal & Implant Science, 44(1), 25–32. 24616831 10.5051/jpis.2014.44.1.25 PMC3945394

[cre2736-bib-0010] Van Dyke, T. E. (2017). Pro‐resolving mediators in the regulation of periodontal disease. Molecular Aspects of Medicine, 58, 21–36. 28483532 10.1016/j.mam.2017.04.006 PMC5660638

[cre2736-bib-0011] Van Dyke, T. E. , & Serhan, C. N. (2003). Resolution of inflammation: A new paradigm for the pathogenesis of periodontal diseases. Journal of Dental Research, 82(2), 82–90. 12562878 10.1177/154405910308200202

[cre2736-bib-0012] Eberhard, J. , Heilmann, F. , Açil, Y. , Albers, H. K. , & Jepsen, S. (2002). Local application ofn−3 orn−6 polyunsaturated fatty acids in the treatment of human experimental gingivitis: Treatment of experimental gingivitis by polyunsaturated fatty acids. Journal of Clinical Periodontology, 29(4), 364–369. 11966935 10.1034/j.1600-051x.2002.290413.x

[cre2736-bib-0014] Elgendy, E. A. , & Kazem, H. H. (2018). Effect of Omega‐3 fatty acids on chronic periodontitis patients in postmenopausal women: A randomised controlled clinical study. Oral Health & Preventive Dentistry, 16(4), 327–332. 30175329 10.3290/j.ohpd.a40957

[cre2736-bib-0016] El‐Sharkawy, H. , Aboelsaad, N. , Eliwa, M. , Darweesh, M. , Alshahat, M. , Kantarci, A. , Hasturk, H. , & Van Dyke, T. E. (2010). Adjunctive treatment of chronic periodontitis with daily dietary supplementation with omega‐3 fatty acids and low‐dose aspirin. Journal of Periodontology, 81(11), 1635–1643. 20572767 10.1902/jop.2010.090628

[cre2736-bib-0017] Elwakeel, N. M. , & Hazaa, H. H. (2015). Effect of omega 3 fatty acids plus low‐dose aspirin on both clinical and biochemical profiles of patients with chronic periodontitis and type 2 diabetes: A randomized double blind placebo‐controlled study. Journal of Periodontal Research, 50(6), 721–729. 25604769 10.1111/jre.12257

[cre2736-bib-0018] Farhad, S. Z. , Amini, S. , Mahdian, A. , Barkatain, M. , & Mafi, M. (2014). Adjunctive low‐dose Aspirin plus Omega‐3 fatty acid versus low‐dose doxycycline on chronic periodontitis. Journal of Islamic Dental Association of IRAN, 26(4), 226–232.

[cre2736-bib-0019] Harris, W. S. (2007). Expert opinion: Omega‐3 fatty acids and bleeding‐cause for concern? The American Journal of Cardiology, 99(6a), S44–S46. 10.1016/j.amjcard.2006.11.021 17368278

[cre2736-bib-0020] Hasturk, H. , Kantarci, A. , Ohira, T. , Arita, M. , Ebrahimi, N. , Chiang, N. , Petasis, N. A. , Levy, B. D. , Serhan, C. N. , & Van Dyke, T. E. (2006). RvE1 protects from local inflammation and osteoclast‐mediated bone destruction in periodontitis. The FASEB Journal, 20(2), 401–403. 16373400 10.1096/fj.05-4724fje

[cre2736-bib-0021] Heitz‐Mayfield, L. J. A. , & Lang, N. P. (2013). Surgical and nonsurgical periodontal therapy. Learned and unlearned concepts. Periodontology 2000, 62(1), 218–231. 23574468 10.1111/prd.12008

[cre2736-bib-0022] Heo, H. , Bae, J. H. , Amano, A. , Park, T. , & Choi, Y. H. (2022). Supplemental or dietary intake of omega‐3 fatty acids for the treatment of periodontitis: A meta‐analysis. Journal of Clinical Periodontology, 49(4), 362–377. 10.1111/jcpe.13603 35141945

[cre2736-bib-0023] Higgins, J. P. T. T. J. , Chandler, J. , Cumpston, M. , Li, T. , Page, M. J. , & Welch, V. A. (2019). Cochrane handbook for systematic reviews of interventions version 6.0 (updated July 2019) (Vol. 2019). The Cochrane Collaboration.

[cre2736-bib-0024] Kesavalu, L. , Bakthavatchalu, V. , Rahman, M. M. , Su, J. , Raghu, B. , Dawson, D. , Fernandes, G. , & Ebersole, J. L. (2007). Omega‐3 fatty acid regulates inflammatory cytokine/mediator messenger RNA expression in porphyromonas gingivalis‐induced experimental periodontal disease. Oral Microbiology and Immunology, 22(4), 232–239. 17600534 10.1111/j.1399-302X.2007.00346.x

[cre2736-bib-0025] Keskiner, I. , Saygun, I. , Bal, V. , Serdar, M. , & Kantarci, A. (2017). Dietary supplementation with low‐dose omega‐3 fatty acids reduces salivary tumor necrosis factor‐α levels in patients with chronic periodontitis: A randomized controlled clinical study. Journal of Periodontal Research, 52(4), 695–703. 28177133 10.1111/jre.12434

[cre2736-bib-0026] Kruse, A. B. , Kowalski, C. D. , Leuthold, S. , Vach, K. , Ratka‐Krüger, P. , & Woelber, J. P. (2020). What is the impact of the adjunctive use of omega‐3 fatty acids in the treatment of periodontitis? A systematic review and meta‐analysis. Lipids in Health and Disease, 19(1), 100. 32438906 10.1186/s12944-020-01267-x PMC7240972

[cre2736-bib-0027] Liberati, A. , Altman, D. G. , Tetzlaff, J. , Mulrow, C. , Gotzsche, P. C. , Ioannidis, J. P. A. , Clarke, M. , Devereaux, P. J. , Kleijnen, J. , & Moher, D. (2009). The PRISMA statement for reporting systematic reviews and meta‐analyses of studies that evaluate healthcare interventions: explanation and elaboration. BMJ, 339, b2700. 19622552 10.1136/bmj.b2700 PMC2714672

[cre2736-bib-0028] Martinez, G. L. , Koury, J. C. , Brito, F. , Fischer, R. G. , Gustafsson, A. , & Figueredo, C. M. (2014). The impact of non‐surgical periodontal treatment on serum levels of long chain‐polyunsaturated fatty acids: A pilot randomized clinical trial. Journal of Periodontal Research, 49(2), 268–274. 23721647 10.1111/jre.12104

[cre2736-bib-0029] Michalowicz, B. S. , Diehl, S. R. , Gunsolley, J. C. , Sparks, B. S. , Brooks, C. N. , Koertge, T. E. , Califano, J. V. , Burmeister, J. A. , & Schenkein, H. A. (2000). Evidence of a substantial genetic basis for risk of adult periodontitis. Journal of Periodontology, 71(11), 1699–1707. 11128917 10.1902/jop.2000.71.11.1699

[cre2736-bib-0030] Morrison, E. C. , Ramfjord, S. P. , & Hill, R. W. (1980). Short‐term effects of initial, nonsurgical periodontal treatment (hygienic phase). Journal of Clinical Periodontology, 7(3), 199–211. 7000853 10.1111/j.1600-051x.1980.tb01963.x

[cre2736-bib-0032] Serhan, C. N. , Chiang, N. , & Van Dyke, T. E. (2008). Resolving inflammation: Dual anti‐inflammatory and pro‐resolution lipid mediators. Nature Reviews Immunology, 8(5), 349–361. 10.1038/nri2294 PMC2744593 18437155

[cre2736-bib-0033] Serhan, C. N. , Hong, S. , Gronert, K. , Colgan, S. P. , Devchand, P. R. , Mirick, G. , & Moussignac, R. L. (2002). Resolvins. Journal of Experimental Medicine, 196(8), 1025–1037. 12391014 10.1084/jem.20020760 PMC2194036

[cre2736-bib-0034] Stańdo, M. , Piatek, P. , Namiecinska, M. , Lewkowicz, P. , & Lewkowicz, N. (2020). Omega‐3 polyunsaturated fatty acids EPA and DHA as an adjunct to non‐surgical treatment of periodontitis: A randomized clinical trial. Nutrients, 12(9), 2614. 32867199 10.3390/nu12092614 PMC7551834

[cre2736-bib-0035] Stark, K. D. , Van Elswyk, M. E. , Higgins, M. R. , Weatherford, C. A. , & Salem, N., Jr. (2016). Global survey of the omega‐3 fatty acids, docosahexaenoic acid and eicosapentaenoic acid in the blood stream of healthy adults. Progress in Lipid Research, 63, 132–152. 27216485 10.1016/j.plipres.2016.05.001

[cre2736-bib-0036] Sterne, J. A. C. , Savović, J. , Page, M. J. , Elbers, R. G. , Blencowe, N. S. , Boutron, I. , Cates, C. J. , Cheng, H. Y. , Corbett, M. S. , Eldridge, S. M. , Emberson, J. R. , Hernán, M. A. , Hopewell, S. , Hróbjartsson, A. , Junqueira, D. R. , Jüni, P. , Kirkham, J. J. , Lasserson, T. , Li, T. , … Higgins, J. P. T. (2019). RoB 2: A revised tool for assessing risk of bias in randomised trials. BMJ, 366, l4898. 31462531 10.1136/bmj.l4898

[cre2736-bib-0037] Sun, Y. P. , Oh, S. F. , Uddin, J. , Yang, R. , Gotlinger, K. , Campbell, E. , Colgan, S. P. , Petasis, N. A. , & Serhan, C. N. (2007). Resolvin D1 and its aspirin‐triggered 17R epimer. Journal of Biological Chemistry, 282(13), 9323–9334. 17244615 10.1074/jbc.M609212200

[cre2736-bib-0038] Tomasi, C. , Leyland, A. H. , & Wennström, J. L. (2007). Factors influencing the outcome of non‐surgical periodontal treatment: A multilevel approach. Journal of Clinical Periodontology, 34(8), 682–690. 17635246 10.1111/j.1600-051X.2007.01111.x

[cre2736-bib-0039] Tur, J. A. , Bibiloni, M. M. , Sureda, A. , & Pons, A. (2012). Dietary sources of omega 3 fatty acids: Public health risks and benefits. British Journal of Nutrition, 107(Suppl. 2), S23–S52. 22591897 10.1017/S0007114512001456

