# Supplementary material for: Effects of Omega‐3 as an adjuvant in the treatment of periodontal disease: A systematic review and meta‐analysis
Source: Clin Exp Dent Res. 2023 Jun 21;9(4):545–56. doi: 10.1002/cre2.736 (PMC10441607; doi:10.1002/cre2.736)
Supplement: Supplementary file 1 — Supporting information. [file CRE2-9-545-s001.pdf]

Database	Pub Med	Embase	Cochrane
Population	(chronic periodontitis/exp OR adult periodontitis OR chronic periodontitis OR periodontal disease/exp OR parodontal disease OR periodontal disease OR periodontal attachment loss OR periodontal disease OR periodontal diseases) AND	('chronic periodontitis'/exp OR 'adult periodontitis' OR 'chronic periodontitis' OR 'periodontal disease'/exp OR 'paradontal disease' OR 'peridontal disease' OR 'periodontal attachment loss' OR 'periodontal disease' OR 'periodontal diseases') AND	('chronic periodontitis'/exp OR 'adult periodontitis' OR 'chronic periodontitis' OR 'periodontal disease'/exp OR 'paradontal disease' OR 'peridontal disease' OR 'periodontal attachment loss' OR 'periodontal disease' OR 'periodontal diseases') AND
Intervention/comparison	(fatty acid/exp OR fatty acid OR fatty acids OR unsaturated fatty acid/exp OR fatty acid, unsaturated OR fatty acids, unsaturated OR unsaturated fatty acid OR unsaturated lipid OR omega 3 fatty acid/exp OR omega 3 fatty acid OR fatty acids, omega 3' OR fatty acids, omega-3' OR 'n 3 fatty acid OR 'n 3 polyunsaturated fatty acid OR omega 3' OR omega 3 polyunsaturated fatty acid OR 'omega3 polyunsaturated fatty acid OR pufa OR docosahexaenoic acid/exp OR docosahexaenoic acid OR docosahexaenoic acids OR docosahexaenoic acid OR fish oil/exp OR fish oil OR fish oils OR eicosapentaenoic acid/exp OR omega 3 eicosapentaenoic acid) AND	('fatty acid'/exp OR 'fatty acid' OR 'fatty acids' OR 'unsaturated fatty acid'/exp OR 'fatty acid, unsaturated' OR 'fatty acids, unsaturated' OR 'unsaturated fatty acid' OR 'unsaturated lipid' OR 'omega 3 fatty acid'/exp OR 'omega 3 fatty acid' OR 'fatty acids, omega 3' OR 'fatty acids, omega-3' OR 'n 3 fatty acid' OR 'n 3 polyunsaturated fatty acid' OR 'omega 3' OR 'omega 3 polyunsaturated fatty acid' OR 'omega3 polyunsaturated fatty acid' OR pufa OR 'docosahexaenoic acid'/exp OR 'docosahexaenoic acid' OR 'docosahexaenoic acids' OR 'docosahexenoic acid' OR 'fish oil'/exp OR 'fish oil' OR 'fish oils' OR 'icosapentaenoic acid'/exp OR 'omega 3 eicosapentaenoic acid') AND	('fatty acid'/exp OR 'fatty acid' OR 'fatty acids' OR 'unsaturated fatty acid'/exp OR 'fatty acid, unsaturated' OR 'fatty acids, unsaturated' OR 'unsaturated fatty acid' OR 'unsaturated lipid' OR 'omega 3 fatty acid'/exp OR 'omega 3 fatty acid' OR 'fatty acids, omega 3' OR 'fatty acids, omega-3' OR 'n 3 fatty acid' OR 'n 3 polyunsaturated fatty acid' OR 'omega 3' OR 'omega 3 polyunsaturated fatty acid' OR 'omega3 polyunsaturated fatty acid' OR pufa OR 'docosahexaenoic acid'/exp OR 'docosahexaenoic acid' OR 'docosahexaenoic acids' OR 'docosahexenoic acid' OR 'fish oil'/exp OR 'fish oil' OR 'fish oils' OR 'icosapentaenoic acid'/exp OR 'omega 3 eicosapentaenoic acid')AND
Outcome	(therapy/exp OR therapy OR disease treatment OR diseases treatment OR therapeutic efficacy OR therapy OR treatment efficacy OR intake)	('therapy'/exp OR therapy OR 'disease treatment' OR 'diseases treatment' OR 'therapeutic efficacy' OR 'therapy' OR 'treatment efficacy' OR intake)	('therapy'/exp OR therapy OR 'disease treatment' OR 'diseases treatment' OR 'therapeutic efficacy' OR 'therapy' OR 'treatment efficacy' OR intake)
Additional Filters	(Humans[Mesh] AND English[lang] AND (dietsuppl[sb] OR cam[sb]) AND (medline[sb] OR jsubsetd[text]) AND (adult[MeSH] OR adult[MeSH:noexp] OR aged[MeSH]))	('controlled clinical trial'/de OR 'double blind procedure'/de OR 'intervention study'/de OR 'randomized controlled trial'/de OR 'randomized controlled trial topic'/de OR 'single blind procedure'/de) AND ('docosahexaenoic acid'/dd OR 'fatty acid'/dd OR 'fish oil'/dd OR 'icosapentaenoic acid'/dd OR 'omega 3 fatty acid'/dd) AND ('chronic periodontitis'/dm OR 'periodontal disease'/dm OR 'periodontal pocket'/dm OR 'periodontitis'/dm)	/
